# Computational and Molecular Properties of Starburst Amacrine Cell Synapses Differ With Postsynaptic Cell Type

**DOI:** 10.3389/fncel.2021.660773

**Published:** 2021-07-26

**Authors:** Joseph Pottackal, Joshua H. Singer, Jonathan B. Demb

**Affiliations:** ^1^Interdepartmental Neuroscience Program, Yale University, New Haven, CT, United States; ^2^Department of Biology, University of Maryland, College Park, College Park, MD, United States; ^3^Department of Ophthalmology and Visual Science, Yale University, New Haven, CT, United States; ^4^Department of Cellular and Molecular Physiology, Yale University, New Haven, CT, United States; ^5^Department of Neuroscience, Yale University, New Haven, CT, United States

**Keywords:** GABA, interneurons, neural circuits, optogenetics, parallel processing, retina, synaptic transmission

## Abstract

A presynaptic neuron can increase its computational capacity by transmitting functionally distinct signals to each of its postsynaptic cell types. To determine whether such computational specialization occurs over fine spatial scales within a neurite arbor, we investigated computation at output synapses of the starburst amacrine cell (SAC), a critical component of the classical direction-selective (DS) circuit in the retina. The SAC is a non-spiking interneuron that co-releases GABA and acetylcholine and forms closely spaced (<5 μm) inhibitory synapses onto two postsynaptic cell types: DS ganglion cells (DSGCs) and neighboring SACs. During dynamic optogenetic stimulation of SACs in mouse retina, whole-cell recordings of inhibitory postsynaptic currents revealed that GABAergic synapses onto DSGCs exhibit stronger low-pass filtering than those onto neighboring SACs. Computational analyses suggest that this filtering difference can be explained primarily by presynaptic properties, rather than those of the postsynaptic cells *per se*. Consistent with functionally diverse SAC presynapses, blockade of N-type voltage-gated calcium channels abolished GABAergic currents in SACs but only moderately reduced GABAergic and cholinergic currents in DSGCs. These results jointly demonstrate how specialization of synaptic outputs could enhance parallel processing in a compact interneuron over fine spatial scales. Moreover, the distinct transmission kinetics of GABAergic SAC synapses are poised to support the functional diversity of inhibition within DS circuitry.

## Introduction

Within a neural circuit, divergence permits the activity of one presynaptic cell to influence multiple postsynaptic cell types in parallel. The functional impact of divergence is enhanced if the presynaptic neuron communicates differently to each postsynaptic cell type. For example, the dynamics of transmission (e.g., the characteristics of short-term, use-dependent plasticity) from a presynaptic neuron can vary systematically with the identity of the postsynaptic partner (Muller and Nicholls, 1974; Katz et al., 1993; Davis and Murphey, 1993; Markram et al., 1998; Reyes et al., 1998; Scanziani et al., 1998). Thus, resolving synaptic mechanisms that diversify output signals reveals strategies for information processing within neural circuits.

To investigate synaptic mechanisms for divergent output signals, we leveraged the well-defined connectivity within the direction-selective (DS) circuit of the mature mouse retina. This circuit depends critically on the starburst amacrine cell (SAC), an axon-less, non-spiking interneuron that provides GABAergic inhibition to both neighboring SACs and DS ganglion cells (DSGCs) (Fried et al., 2002; Lee and Zhou, 2006; Kostadinov and Sanes, 2015; Ding et al., 2016), as well as cholinergic excitation to DSGCs but not SACs (Zheng et al., 2004; Lee et al., 2010). Thus, this circuit implements signal divergence at two levels: differences in postsynaptic cell type (i.e., GABAergic SAC→SAC vs. GABAergic SAC→DSGC) and differences in neurotransmitter (i.e., GABAergic vs. cholinergic SAC→DSGC). Previously, we determined that the distinct time courses of GABAergic and cholinergic transmission from SACs to DSGCs can be fully explained by transmitter-specific differences in postsynaptic receptor kinetics (Pottackal et al., 2020). It remains unknown, however, whether GABAergic synapses from SACs onto distinct postsynaptic cell types differ in their computational properties and, if so, whether these differences arise pre- or postsynaptically.

**FIGURE 1:**
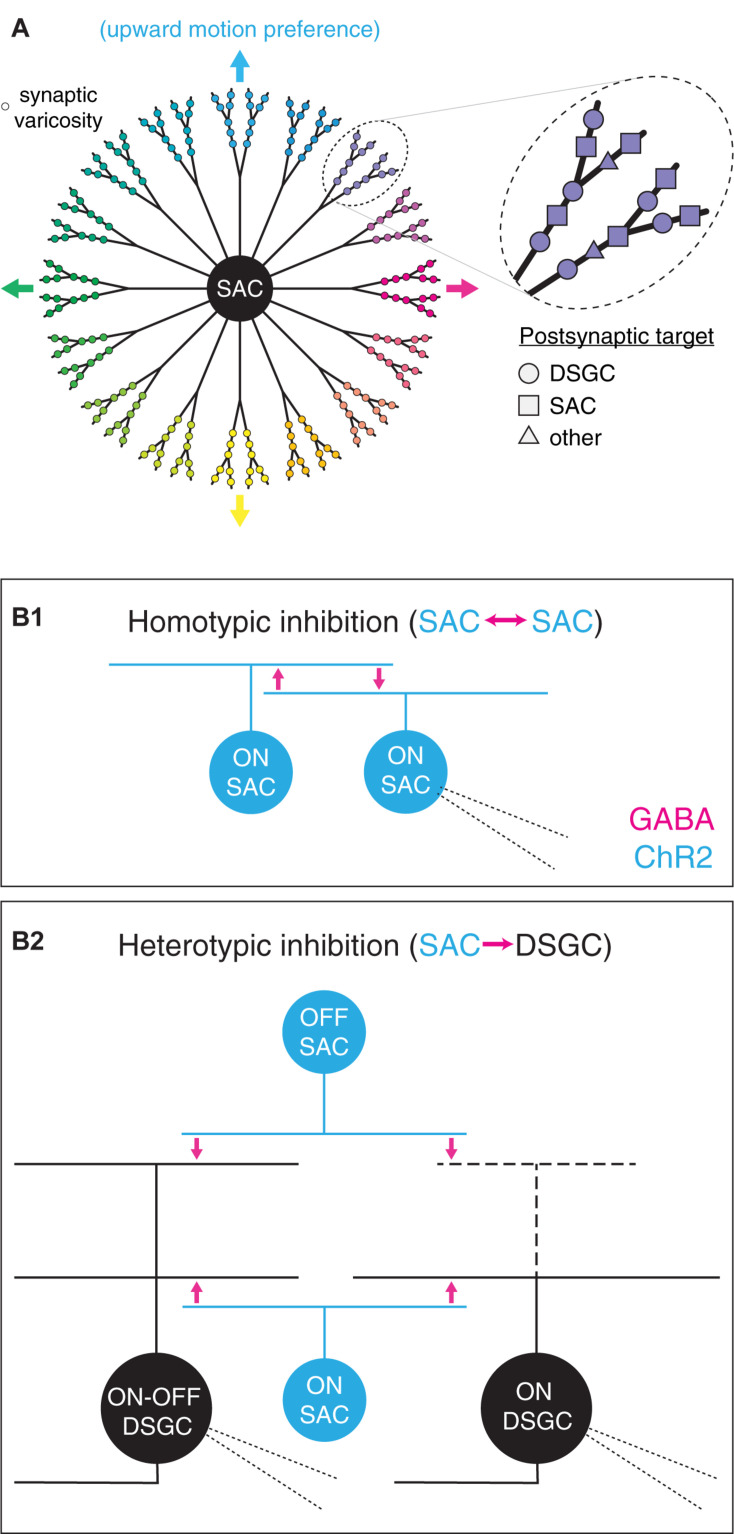
Functional diversity and spatial organization of synaptic outputs in starburst amacrine cells. **(A)** Diverse properties of synaptic varicosities in a SAC. *Left*, functional parsing of SAC neurites into sectors with distinct preferences for direction of motion. Varicosity color indicates preferred direction. Arrows indicate cardinal directions. *Right*, expanded view of varicosities within one neurite sector. Varicosity shape indicates postsynaptic cell type identity. **(B)** An optogenetic approach to comparing GABAergic transmission from SACs to distinct postsynaptic cell types. **(B1)** Homotypic inhibition between SACs. Photostimulation of channelrhodopsin-2-expressing (ChR2^+^) ON SACs evokes reciprocal GABA release onto neighboring ON SACs. **(B2)** Heterotypic inhibition between SACs and DSGCs. Optogenetic stimulation of ON and OFF SACs evokes GABA release onto ON-OFF and ON DSGCs. Thick dashed lines indicate sparse or absent OFF-layer dendrites in ON DSGCs. Fine dashed lines indicate patch pipettes for targeted whole-cell recording.

In addition to targeting diverse postsynaptic partners, the output synapses of SACs exhibit diverse visual response properties that map systematically onto cellular morphology. Specifically, each SAC neurite is depolarized preferentially by centrifugal motion (i.e., motion from the soma toward the distal tip of the neurite; Euler et al., 2002; Hausselt et al., 2007; Chen et al., 2016; Vlasits et al., 2016; Koren et al., 2017; Morrie and Feller, 2018). A radially symmetric SAC arbor is thus functionally organized into >20 sectors, each with a distinct direction preference. Additionally, the distal region of each sector contains a cluster of presynaptic active zones that exhibit locally correlated activity over a scale of tens of micrometers (Poleg-Polsky et al., 2018; Figure 1A). Within a sector, though, output synapses are not spatially segregated according to postsynaptic cell type; indeed, intermingled presynapses onto SACs and DSGCs can be separated by <5 μm (Ding et al., 2016; Figure 1A). Thus, if signal processing at GABAergic SAC synapses differs according to the identity of the postsynaptic cell type (i.e., SAC vs. DSGC), functional diversity among SAC outputs may exist on an even finer spatial scale than that defined by activity correlations (Poleg-Polsky et al., 2018). Indeed, GABAergic inhibition from SACs appears to subserve different functions in postsynaptic DSGCs and SACs. In DSGCs, inhibition persists in order to coincide with and counter excitation during null-direction motion (reviewed in Vaney et al., 2012); by contrast, inhibition in SACs precedes excitation, thereby relieving synaptic depression at output synapses onto DSGCs (Chen et al., 2020).

To test the hypothesis that SAC output synapses form parallel channels that differ functionally with postsynaptic cell identity, we compared the temporal response characteristics of divergent GABAergic outputs from SACs by combining optogenetics, electrophysiology, and computational analyses. Strikingly, we found that low-pass filtering was more pronounced at GABAergic synapses onto DSGCs than at those onto SACs. Furthermore, this temporal difference between GABAergic SAC synapses appeared to be generated predominantly by presynaptic mechanisms. Thus, a SAC generates parallel GABAergic outputs that differ functionally between postsynaptic cell types, which may support the apparently distinct roles of GABAergic SAC synapses at two loci within DS circuitry.

## Materials and Methods

### Animals

All animal procedures were approved by the Institutional Animal Care and Use Committee at Yale University and were in compliance with National Institutes of Health guidelines. Mice of both sexes were maintained on a C57BL/6 background and studied between postnatal days 28 and 90. All experimental animals were generated by crossing homozygous *Chat-ires-cre* mice (B6;129S6-Chat^*tm2(cre)Lowl*^/J; The Jackson Laboratory #006410) with homozygous *Ai32* mice [Madisen et al., 2012; B6.Cg-Gt(ROSA)26Sor^*tm32(CAG–COP4* × *H134R/EYFP)Hze*^/J; The Jackson Laboratory #024109] to yield offspring that were heterozygous for both transgenes. In retinas of these mice, Cre expression is driven by endogenous *Chat* regulatory elements and induces selective expression of a channelrhodopsin-2 (ChR2)/enhanced yellow fluorescent protein (EYFP) fusion protein in ON and OFF SACs.

### Electrophysiology

Mice were euthanized following ∼1 h of dark adaptation. Subsequently, both eyes were enucleated and placed in a dissection dish containing Ames medium (A1420, MilliporeSigma) supplemented with 22.6 mM NaHCO_3_ (MilliporeSigma) and bubbled with 95% oxygen/5% carbon dioxide gas at room temperature. Retinas were dissected under infrared illumination using stereomicroscope-mounted night vision goggles (B.E. Meyers). After removal of the retina from the eyecup, the vitreous humor was stripped away and a single relaxing cut was made along the nasotemporal axis. Retinas were then affixed to mixed cellulose ester filter membranes (HAWP01300, MilliporeSigma) and kept at room temperature until recording. Before recording, filter-mounted retinas were transferred to a custom recording chamber and fastened by a tissue harp. During recording, the chamber was perfused with Ames medium at a flow rate of 4–6 mL/min and a temperature of 32–34°C.

For whole-cell recordings, patch pipettes were pulled from borosilicate glass capillaries (1B120F-4, World Precision Instruments) and had tip resistances of 4–6 MΩ for ganglion cell recordings or 5–8 MΩ for amacrine cell recordings. Patch pipettes were back-filled with internal solutions containing the following (in mM): 120 Cs-methanesulfonate, 5 TEA-Cl, 10 HEPES, 10 BAPTA, 3 NaCl, 2 QX-314-Cl, 4 ATP-Mg, 0.4 GTP-Na_2_, and 10 phosphocreatine-tris_2_, at pH 7.3 and 280 mOsm for voltage-clamp recordings; or 120 K-methanesulfonate, 10 HEPES, 0.1 EGTA, 5 NaCl, 4 ATP-Mg, 0.4 GTP-Na_2_, and 10 phosphocreatine-tris_2_, at pH 7.3 and 280 mOsm for current-clamp recordings. All compounds in internal solutions were obtained from MilliporeSigma. During all recordings, membrane current or voltage was amplified (MultiClamp 700B, Axon Instruments), digitized at 5 or 10 kHz (Digidata 1440A, Molecular Devices), and recorded (pClamp 10.0, Molecular Devices). During voltage-clamp recordings, inhibitory or excitatory currents were isolated by clamping at the reversal potentials for cations (E_cation_; ∼0 mV) or chloride (E_Cl_; ∼−67 mV), respectively. Series resistance (10–25 MΩ) was compensated by 50%, and recordings were corrected for a −9-mV liquid junction potential.

Direction-selective ganglion cells were initially identified by obtaining loose-patch spike recordings of visual responses in unlabeled GCs. Visual stimuli were displayed by a modified video projector (λ_peak_ = 395 nm) focused through a sub-stage condenser lens onto the retina (Borghuis et al., 2013, 2014). Mean luminance was ∼10^4^ photoisomerizations cone^–1^ s^–1^ (Borghuis et al., 2014). Putative ON-OFF DSGCs and ON DSGCs were first identified and differentiated according to their distinct spike responses to a light spot (5 s, 400-μm diameter) of positive contrast: ON-OFF DSGCs fired transiently at stimulus onset and offset, whereas ON DSGCs fired in a sustained manner over the duration of the stimulus (Weng et al., 2005; Sun et al., 2006; Dhande et al., 2013). Most putative DSGCs were also presented with drifting gratings to confirm DS spike responses (Park et al., 2014). After establishment of a voltage-clamp recording, DSGC identity was confirmed by the presence of both inhibitory postsynaptic currents (IPSCs) (GABAergic) and excitatory postsynaptic currents (EPSCs) (cholinergic) during optogenetic stimulation of SACs (Sethuramanujam et al., 2016; Hanson et al., 2019; Pottackal et al., 2020). ON SACs were identified by visualizing EYFP^+^ somata in the ganglion cell layer using a custom-built two-photon laser-scanning microscope controlled by ScanImage (Vidrio Technologies) (Borghuis et al., 2013). Two-photon excitation was provided by a tunable Coherent Chameleon Ultra II laser (λ_peak_ = 910 nm).

Optogenetic stimulation of ChR2^+^ SACs was performed using an LED (λ_peak_ = 470 nm; M470L3, Thorlabs) projected through the aperture (400-μm diameter) of an iris diaphragm (CP20S, Thorlabs), driven by a T-Cube LED driver (LEDD1B, Thorlabs), and focused through a sub-stage condenser lens onto the retina. The maximum light intensity (Φ_max_) at the sample plane was 4.8 × 10^17^ quanta (Q) cm^–2^ s^–1^. Stimuli were corrected for a nonlinear relationship between voltage input to the LED driver and light output of the LED, which was measured at the sample plane. Rod- and cone-mediated inputs were silenced by supplementing the bath solution with the following compounds (in μM): 50 D-AP5 (Alomone), 50 DNQX (Alomone), 20 L-AP4 (Alomone), and 2 ACET (Tocris) (Park et al., 2015, 2018, 2020; Pottackal et al., 2020, 2021). During some experiments, N-type voltage-gated calcium channels (VGCCs) were also blocked by adding 0.3 μM ω-conotoxin G6A (Alomone) to the solution described above. For these experiments, both the control and experimental solutions were supplemented with 0.01% cytochrome C (MilliporeSigma) to reduce non-specific adhesion of the peptide antagonist to plastic tubing and glassware. For experiments in which extracellular Ca^2+^ was varied, Ames medium was replaced by a Ringer solution consisting of the following (in mM): 119 NaCl, 23 NaHCO_3_, 10 glucose, 2.5 KCl, 2 Na-(L)-lactate, 2 Na-pyruvate, 1.5 Na_2_SO_4_, and 1.25 NaH_2_PO_4_. CaCl_2_ and MgCl_2_ were variably added to the Ringer solution at a fixed total molarity of 4 mM (e.g., 0.5 mM CaCl_2_ and 3.5 mM MgCl_2_; Jarsky et al., 2010). All compounds included in the Ringer solution were obtained from MilliporeSigma.

### Linear-Nonlinear Cascade Analysis

Linear-nonlinear (LN) cascade analysis was performed as described in detail previously (Jarsky et al., 2011; Pottackal et al., 2020, 2021). Briefly, quasi-white-noise (WN) stimuli were generated by repeated draws from a standard normal distribution and then ideally low-pass filtered at 30 Hz. WN stimuli comprised 10 consecutive 10-s trials, each consisting of 7.5 s of a unique stimulus sequence followed by 2.5 s of a repeated stimulus sequence. For each cell, responses to unique stimuli were used to construct the model, while responses to repeated stimuli were used to assess the accuracy of the model. Trial-to-trial response reliability was measured by computing the Pearson correlation coefficient between each trial’s repeat response and the average of all other trials’ repeat responses and, subsequently, averaging all 10 resulting values. Recordings with response reliability exceeding 0.7 were analyzed further.

**FIGURE 2:**
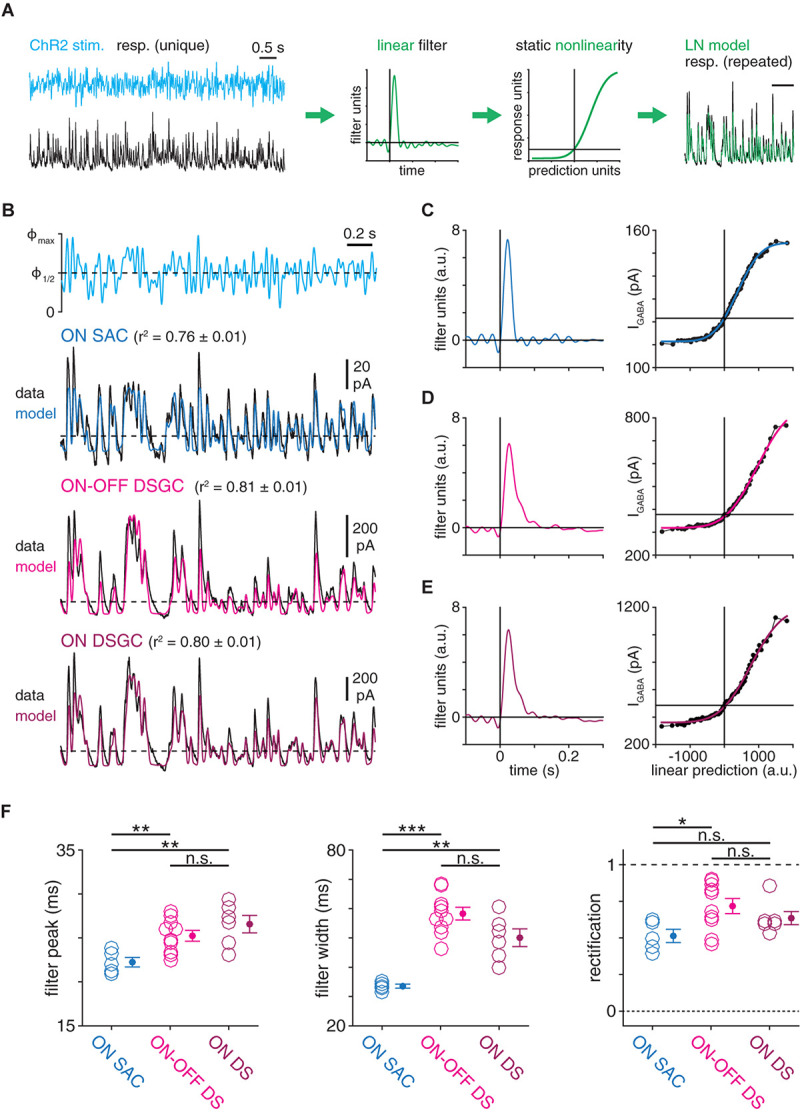
Computational properties of GABAergic SAC synapses differ with postsynaptic cell type identity. **(A)** Schematic illustrating generation of a linear-nonlinear (LN) model from a response evoked by white-noise (WN) ChR2 stimulation (see “Materials and Methods section”). **(B)** Linear-nonlinear models obtained from IPSCs recorded in ON SACs and DSGCs during optogenetic white-noise stimulation of SACs. Black traces indicate averaged response to 10 stimulus repeats [maximum stimulus intensity (Φ_max_) = 4.8 × 10^17^ Q cm–^–2^ s^–1^). Colored traces show LN model output. Dashed horizontal lines indicate the response at a linear prediction of 0 (see **C–E**). **(C–E)**, Linear filter (*left*) and static nonlinearity (*right*) obtained from ON SAC **(C)**, ON-OFF DSGC **(D)**, and ON DSGC **(E)** in **(B)**. Horizontal line overlaid on each static nonlinearity indicates the response at a linear prediction of 0. **(F)** Measurements of LN model components obtained from IPSCs recorded in ON SACs (*n* = 5), ON-OFF DSGCs (*n* = 10), and ON DSGCs (*n* = 6). The ON-OFF DSGC shown in **(B,D)** and data from DSGCs in **(F)** are re-plotted from Pottackal et al. (2020). n.s., not significant; *p < 0.05; **p < 0.01; and ***p < 0.001.

To construct an LN model from a recorded response to WN stimulation, a linear filter was first computed by cross-correlating the WN stimulus with the response. Filter width was measured as the full width at 25% of the maximum. For a subset of linear filters (see Figure 7), a biphasicity index *b*_φ_ was measured as:

$$b_{\varphi }=1-\frac{f_{m\,a\,x}-f_{m\,i\,n}}{f_{m\,a\,x}+f_{m\,i\,n}}=2\,\frac{f_{m\,i\,n}}{f_{m\,a\,x}+f_{m\,i\,n}}$$

where *f*_*max*_ = max[*f*(*t*)] and *f*_*min*_ = |min{0,min[*f*(*t*)]}| for 0 < *t* ≤ 60 ms. The filter was then convolved with the stimulus to generate a linear prediction of the response, which was then plotted against the recorded response for each time point. Plotted points were equally divided into 100 bins along the linear prediction axis. Points within each bin were averaged along both dimensions (i.e., predicted and recorded response axes) to generate 100 points, which were then fit to a Gaussian cumulative distribution function *N*(*x*) that acted as the static nonlinearity component of the model. A rectification index *i*_*rect*_ was computed from *N*(*x*) to measure the nonlinearity of each modeled response:

$$i_{r\,e\,c\,t}=\frac{\left|N\,\left[\text{max}\,\left(r_{L\,\left[b\,i\,n\right]}\right)\right]+N\,\left[\text{min}\,\left(r_{L\,\left[b\,i\,n\right]}\right)\right]-2\,N\,\left(0\right)\right|}{N\,\left[\text{max}\,\left(r_{L\,\left[b\,i\,n\right]}\right)\right]-N\,\left[\text{min}\,\left(r_{L\,\left[b\,i\,n\right]}\right)\right]}$$

where *r*_*L[bin]*_ is the set of 100 values obtained after binning and averaging along the linear prediction axis. Finally, the linear prediction was passed through this static nonlinearity to generate the output of the LN model. The accuracy of the model was measured as the squared Pearson correlation coefficient (*r*^2^) between (1) the model’s response to the repeated stimulus and (2) the mean of 10 recorded responses to the repeated stimulus. For all conditions studied using LN analysis, these *r*^2^ values are reported in the corresponding figures. IPSCs recorded from ON-OFF DSGCs during WN stimulation of SACs (Figures 2, 4, 6–8) were included in an earlier study (Pottackal et al., 2020).

### IPSC Contamination Analysis

**FIGURE 3:**
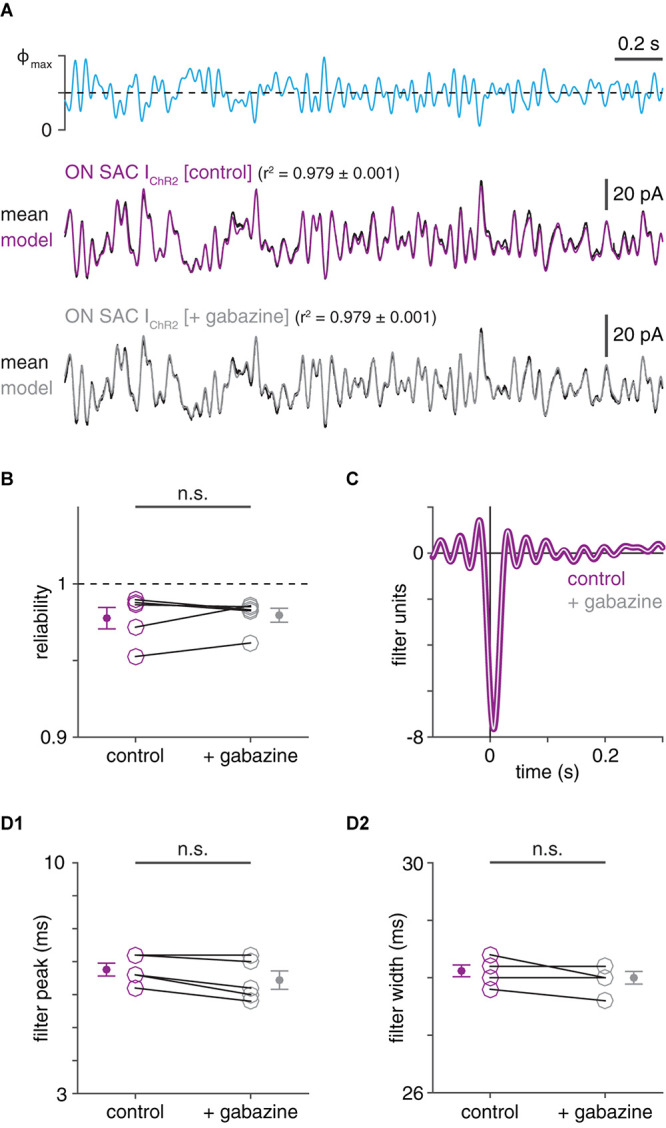
Effective isolation of ChR2- from GABA_A_R-mediated currents in ON SACs using voltage clamp. **(A)** ChR2-mediated inward currents recorded in a ChR2^+^ ON SAC before and after GABA_A_R blockade. *Top*, optogenetic white-noise stimulus (Φ_max_ = 4.8 × 10^17^ Q cm^–2^ s^–1^). ChR2-mediated inward currents before (*middle*) and after (*bottom*) bath application of gabazine (50 μM). **(B)** Inter-trial reliability of ChR2-mediated currents in ON SACs (*n* = 5) before and after gabazine application. **(C)** Linear filters obtained from ChR2-mediated currents in ON SAC shown in **(A)** before (thick purple trace) and after (thin gray trace) gabazine application. **(D)** Peak times **(D1)** and widths **(D2)** of linear filters obtained from LN analysis of ChR2-mediated currents in ON SACs before and after gabazine application. n.s., not significant.

An analysis was performed to evaluate the potential impact of unclamped ChR2 current (I_ChR2_) on IPSCs recorded from a ChR2^+^ SAC clamped at the nominal E_cation_. Traces used in the simulation were averages of 10 recorded responses to the repeated WN sequence. Estimates of unclamped ChR2 current at E_cation_ were constructed by first averaging ChR2 current recorded in 5 ON SACs clamped at E_Cl_ during the repeated WN sequence (25 μM gabazine; Figure 3). To estimate the fractional reduction of I_ChR2_ at E_cation_, the amplitude (10 ms after stimulus onset) of a saturated I_ChR2_ was measured at E_cation_ (−8.4 ± 1.7 pA; *n* = 8 cells) and E_Cl_ (−96.3 ± 7.6 pA; *n* = 3 cells). The ratio of these values (0.088 ± 0.019) provides an estimate of unclamped ChR2 current at E_cation_, from which we derived a conservative scaling factor of 0.146 (mean + 3 × SEM), i.e., an upper bound for the contamination. The mean I_ChR2_ trace was then multiplied by this factor to generate an estimate of the unclamped current at E_cation_. To reflect potential low-pass filtering due to cable properties of SAC neurites, the downscaled I_ChR2_ trace was convolved with an exponential filter characterized by one of four time constants of decay (τ_decay_ = 10, 31.6, 100, or 316 ms). Filters were normalized to have an integral of 1.

**FIGURE 4:**
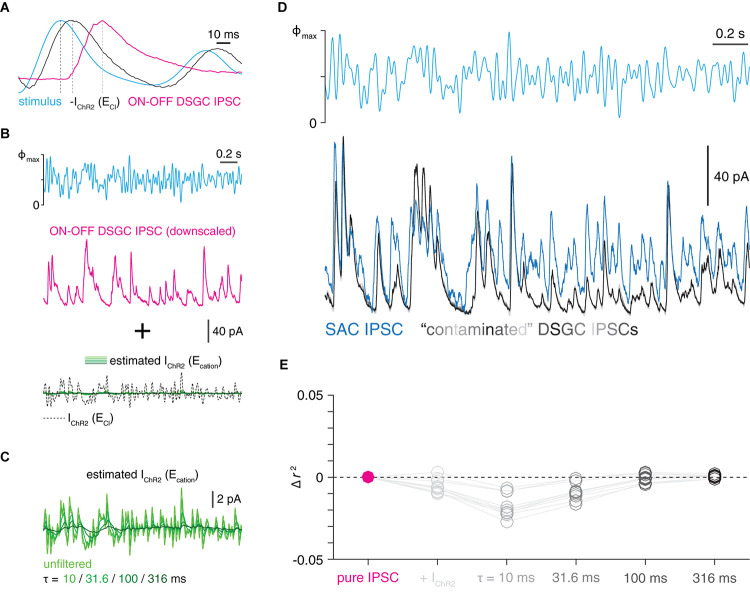
Contamination of IPSCs by unclamped ChR2 current in voltage-clamped SACs cannot explain relatively fast transmission kinetics. **(A)** Comparison of time courses of optogenetic white-noise stimulus sequence (cyan), inverted ChR2 current (black), and IPSC recorded in an ON-OFF DSGC. Gray lines indicate relative peak times for each trace. Each trace is normalized to its peak. ChR2 current trace is the mean of recordings from 5 ON SACs voltage-clamped at E_Cl_ during GABA_A_ receptor blockade. **(B)** Simulating contamination of an IPSC by unclamped ChR2 current. *Top*, optogenetic white-noise stimulus (Φ_max_ = 4.8 × 10^17^ Q cm^–2^ s^–1^). *Middle*, IPSC (average of 10 repeated trials) obtained from an ON-OFF DSGC downscaled to match amplitude of an ON SAC IPSC. *Bottom*, family of ChR2 currents generated by downscaling ChR2 current measured at E_Cl_ and applying a family of exponential filters (see section “Materials and Methods”). Resulting ChR2 current traces serve as estimates of ChR2 current at E_cation_, which are added to the IPSC to simulate contamination. **(C)** Expanded view of contaminating ChR2 currents shown in **(B)**. **(D)** Comparison of SAC IPSCs and “contaminated” IPSCs. *Top*, optogenetic white-noise stimulus (Φ_max_ = 4.8 × 10^17^ Q cm^–2^ s^–1^). *Bottom*, optogenetically evoked IPSCs recorded in an ON SAC (blue trace; mean of 10 trials) overlaid with a simulated series of “contaminated” IPSCs (gray traces) generated using an ON-OFF DSGC IPSC. **(E)** Average changes in squared Pearson correlation coefficients produced by applying the contamination procedure shown in **(B)**. The contamination of a DSGC’s IPSC did not increase its correlation with a SAC’s IPSC (i.e., a positive change in *r*^2^ value); indeed, the opposite was true in most cases.

**FIGURE 5:**
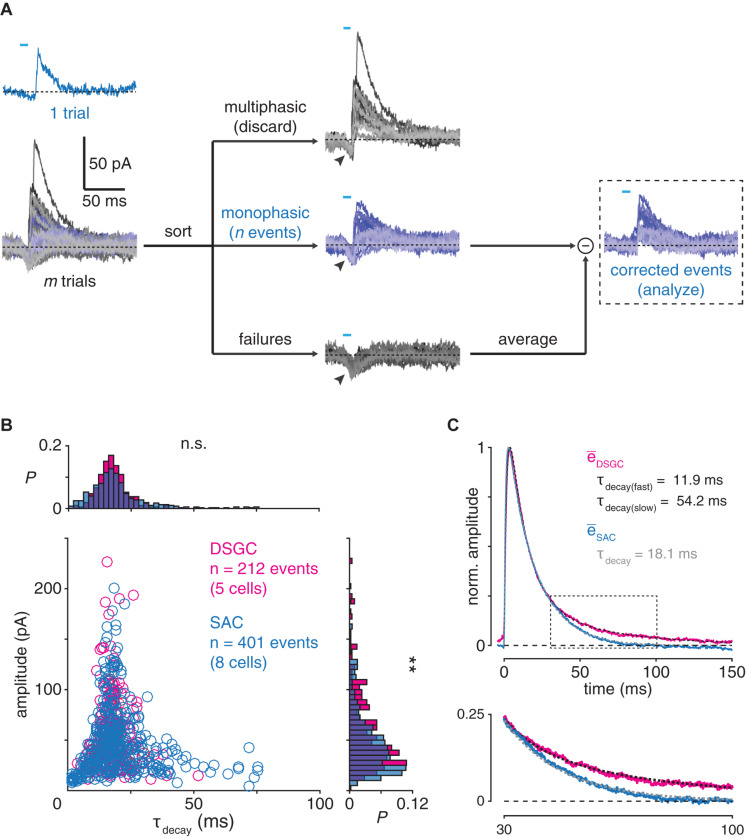
Postsynaptic filtering at GABAergic SAC synapses. **(A)** Generation of evoked monophasic inhibitory postsynaptic currents (emIPSCs) in an ON SAC. Brief (<10 ms) optogenetic stimulation of presynaptic SACs evokes a small, monophasic IPSC (blue). Following *m* trials, *n* monophasic events are distinguished from multiphasic events and failures (gray). Arrowheads indicate unclamped ChR2-mediated photocurrents, which are removed by subtracting the mean of all failures from each emIPSC. **(B)** Comparison of emIPSCs recorded in DSGCs and ON SACs. Amplitude plotted against decay time constant (τ_decay_) for emIPSCs recorded in DSGCs (*n* = 212 events from 4 ON-OFF DSGCs and 1 ON DSGC) and ON SACs (*n* = 401 events from 8 cells). Marginal probability distributions of emIPSC amplitude and τ_decay_ are shown at right and above, respectively. **(C)** Averaged time courses of emIPSCs recorded in DSGCs and ON SACs. *Top*, average of all emIPSCs recorded in DSGCs (${\bar{\text{e}}}_{\text{DSGC}}$, magenta trace) is fit by a function (dashed black trace; see section “Materials and Methods”) with two exponential decay terms (τ_decay(fast)_ = 11.9 ms; τ_decay(slow)_ = 54.2 ms). Overlaid, the average of all emIPSCs recorded in SACs ($\bar{\text{e}}$_SAC_, blue trace) is fit by a similar function with a single exponential decay term (dashed gray trace; τ_decay_ = 18.1 ms). All traces are normalized to their respective maxima. *Bottom*, expanded view of boxed period above. Data from DSGCs is re-plotted from Pottackal et al. (2020). n.s., not significant; **p < 0.01.

**FIGURE 6:**
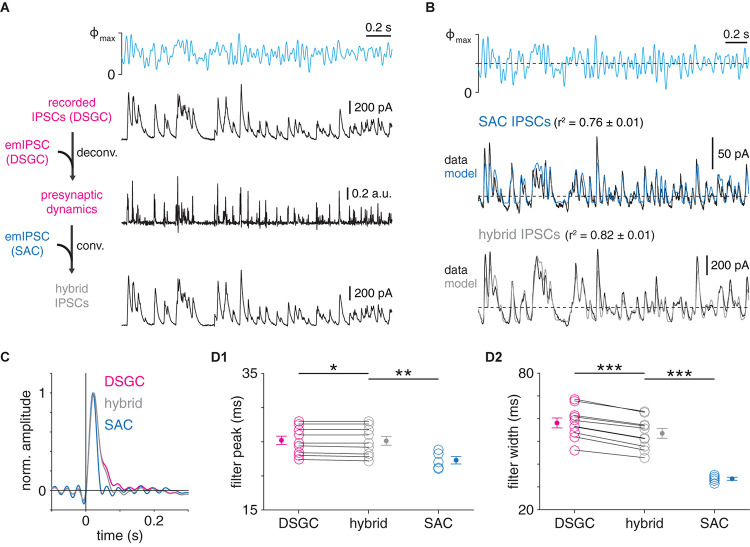
Postsynaptic dynamics only partially explain postsynaptic cell type-specific filtering at GABAergic SAC synapses. **(A)** Protocol for generation of hybrid IPSCs. Wiener deconvolution of ON-OFF DSGC IPSCs removes the contribution of postsynaptic dynamics (DSGC emIPSC) to estimate presynaptic release dynamics. The result is convolved with an estimate of SAC→SAC postsynaptic dynamics (SAC emIPSC) to generate hybrid IPSCs. **(B)** Comparison of recorded and corresponding hybrid IPSC responses to optogenetic WN stimulation of SACs. *Top*, optogenetic WN stimulus. Maximum light intensity (Φ_max_), 4.8 × 10^17^ Q cm^–2^ s^–1^. Mean responses (black) and LN models (colored) for IPSCs recorded in an ON SAC (*top*) and hybrid IPSCs combining (1) presynaptic dynamics of GABAergic transmission to the same ON-OFF DSGC as above with (2) postsynaptic dynamics of GABAergic transmission to ON SACs (*bottom*). **(C)** Comparison of linear filters from recorded and hybrid IPSCs. *Left*, linear filters obtained from GABAergic IPSCs recorded in ON-OFF DSGC (magenta) and ON SAC (blue) in **(B)**, overlaid with linear filter from corresponding hybrid IPSCs shown in **(B)** (gray). **(D)** Summary of filter peak times **(D1)** and widths **(D2)** from recorded and hybrid IPSCs in ON-OFF DSGCs (*n* = 10 cells) and ON SACs (*n* = 5 cells). Data from DSGCS are re-plotted from Pottackal et al. (2020). *p < 0.05; **p < 0.01; and ***p < 0.001.

**FIGURE 7:**
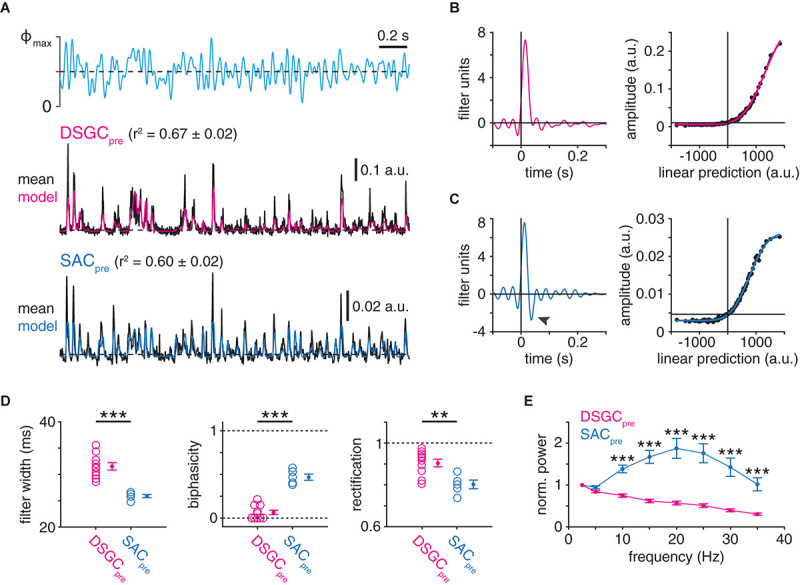
Postsynaptic cell type-specific filtering at GABAergic SAC presynapses. **(A)** LN analysis of deconvolved presynaptic dynamics of GABAergic SAC synapses (see Figure 5A). *Top*, optogenetic stimulus. Maximum light intensity (Φ_max_), 4.8 × 10^17^ Q cm^–2^ s^–1^. *Middle*, presynaptic dynamics isolated from IPSCs recorded in an ON-OFF DSGC (DSGC_pre_). Black trace shows averaged response to 10 stimulus repeats. LN model output for the same sequence is overlaid (red). *Bottom*, same as *middle* for IPSCs recorded in an ON SAC (SAC_pre_; blue, LN model output). **(B)** Linear filter (*left*) and static nonlinearity (*right*) obtained from SAC→ON-OFF DSGC presynaptic dynamics shown in **(A)**. **(C)** Same as **B** for SAC→SAC presynaptic dynamics shown in **(A)**. Black arrowhead indicates negative lobe of linear filter. **(D)** Measurements of LN model components for presynaptic dynamics from IPSCs recorded in ON-OFF DSGCs (*n* = 10) or ON SACs (*n* = 5): filter width (*left*), filter biphasicity index (*middle*), and rectification of nonlinearity (*right*). **(E)** Frequency analysis of isolated presynaptic dynamics estimated from IPSC recordings of ON-OFF DSGCs and ON SACs. Power spectra are normalized to the power at 2.5 Hz and reflect the entire 100-s recording. **p < 0.01; ***p < 0.001.

**FIGURE 8:**
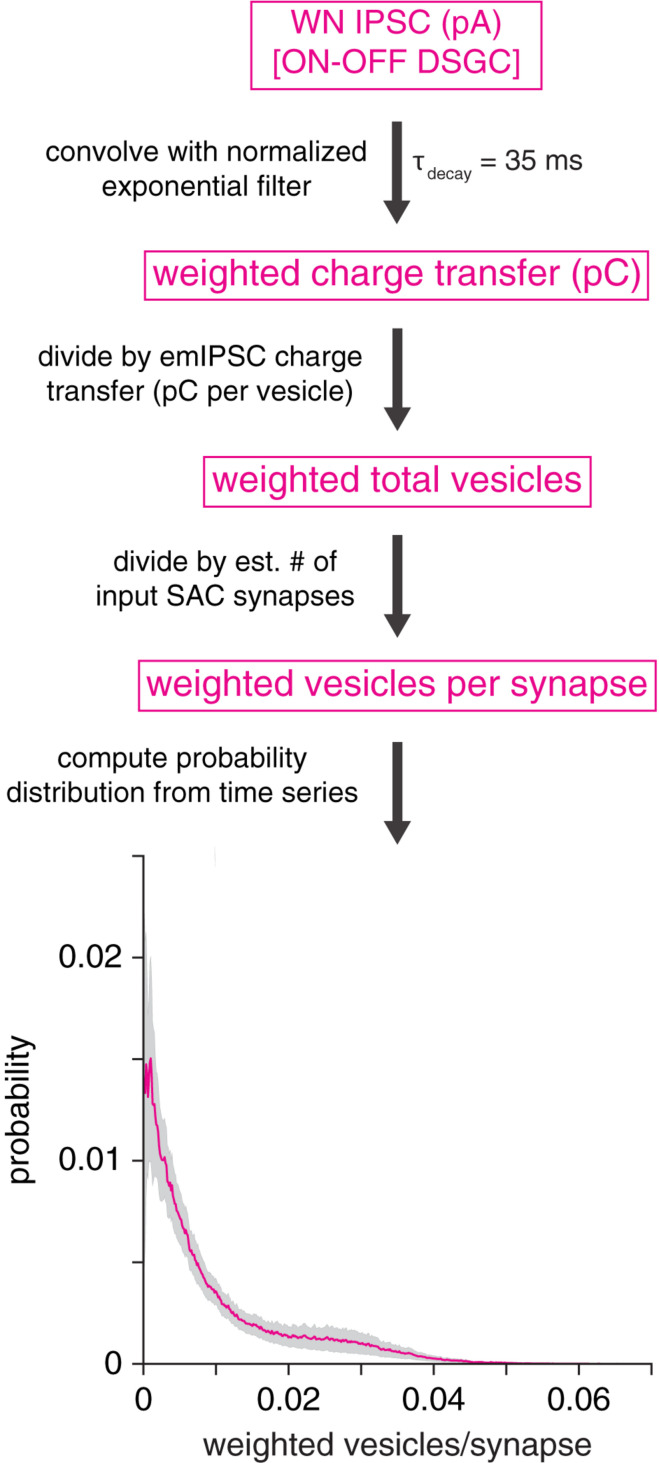
GABA_A_ receptor desensitization is unlikely to explain prolonged IPSCs in DSGCs. Analytical procedure for estimating the average number of vesicles driving fast GABA_A_ receptor desensitization at a SAC synapse onto an ON-OFF DSGC. *Top*, an IPSC is convolved with an exponential filter (peak-normalized), which approximates the time course of fast (τ = 35 ms) GABA_A_ receptor desensitization. Subsequent operations generate a probability distribution of vesicles per synapse. *Bottom*, average probability distribution for estimated number of vesicles driving fast desensitization at a GABAergic SAC synapse onto an ON-OFF DSGC (mean of 10 cells). Shaded area represents ± SEM.

The next stage of the analysis tested the hypothesis that the relatively fast waveforms of SAC IPSCs, measured in response to WN stimulation of SACs, resulted from slow IPSC waveforms (identical to those measured in ON-OFF DSGCs) that were contaminated with an unclamped ChR2 current. To test this, we took all possible pairs of IPSCs recorded in one SAC (*n* = 5 cells) and one ON-OFF DSGC (*n* = 10 cells) and derived a separate scale factor that was computed as the ratio of the standard deviation of each recording (5 × 10 = 50 total scale factors). Each scale factor was used to downscale the ON-OFF DSGC IPSC to have a standard deviation equal to that of the SAC IPSC. The downscaled trace was then summed with one of five variants of the I_ChR2_ trace obtained above: either the unfiltered trace or one of the four filtered traces (5 × 10 × 5 = 250 “contaminated” DSGC IPSCs). The squared Pearson correlation coefficient (*r*^2^) was then computed between the corresponding ON SAC IPSC and each contaminated DSGC IPSC to measure the similarity of each pair of waveforms. This procedure was performed for each combination of SAC IPSC, DSGC IPSC, and I_ChR2_ variant (5 × 10 × 5 = 250 *r*^2^ values). These values were finally averaged across the 5 SACs to yield the 50 values shown in Figure 4E. This procedure evaluated whether contaminating a DSGC IPSC with a ChR2 current could increase its similarity to a measured SAC IPSC.

### Event Analysis

Evoked monophasic IPSCs (emIPSCs) were elicited in a DSGC or an ON SAC by brief (<10 ms) optogenetic stimulation of SACs, as reported previously (Pottackal et al., 2020). Briefly, stimulus intensity and duration were adjusted for each cell so that approximately one-third of trials failed to evoke an IPSC. For each trial, the trace was filtered and thresholded to detect rapid rising phases indicative of evoked IPSCs. An event with exactly one suprathreshold rising phase was identified as a monophasic IPSC. In some ChR2^+^ ON SACs, emIPSCs were partially contaminated by an unclamped inward ChR2 current. In such cases, “failed” trials (i.e., those with no detectable IPSC) were averaged together to isolate the ChR2 current, which was then subtracted from each individual emIPSC recorded in the same cell. For an individual emIPSC, amplitude was measured at its peak, and time constant of decay (τ_decay_) was measured by fitting an exponential function to its decay phase. Prior to averaging, emIPSCs were aligned to the first point at which the amplitude of each filtered event exceeded the detection threshold. For DSGCs and ON SACs, the averages of all aligned emIPSCs were computed separately and fit with functions of the following forms:

$$f_{D\,S\,G\,C}\,\left(t\right)=\left(a_{1}\,e^{-\frac{t}{\tau_{1}}}+a_{2}\,e^{-\frac{t}{\tau_{2}}}\right)\,\left(1-a_{3}\,e^{-\frac{t}{\tau_{3}}}\right)$$

$$f_{S\,A\,C}\,\left(t\right)=\left(a_{1}\,e^{-\frac{t}{\tau_{1}}}\right)\,\left(1-a_{2}\,e^{-\frac{t}{\tau_{2}}}\right)$$

where *a_i_* are amplitude-scaling constants and τ_*i*_ are time constants. emIPSCs from DSGCs were included in an earlier study (Pottackal et al., 2020).

### Wiener Deconvolution

As described previously (Pottackal et al., 2020), Wiener deconvolution was used to isolate presynaptic dynamics from IPSCs measured in either ON-OFF DSGCs or ON SACs during optogenetic WN stimulation of SACs. Briefly, a recorded IPSC was deconvolved with the average emIPSC corresponding to its cell type. A noise estimate, which is essential for Wiener deconvolution, was obtained from a 1-s sample of the pre-stimulus baseline. In net, this procedure yielded a times series consisting of delta function-like events, scaled in amplitude, which putatively correspond to bursts of vesicle release from presynaptic neurons (James et al., 2019). These time series were either: (1) convolved with the emIPSC of the opposing cell type to generate hybrid IPSCs (Figure 6), which were then analyzed; or (2) analyzed directly (Figure 7). A key assumption of this procedure is approximately linear summation of unitary emIPSCs, which is supported by the weak correlations between amplitude and decay time constant in emIPSCs recorded in DSGCs (Kendall’s τ coefficient = 0.025, *p* = 0.596; Figure 5B) and ON SACs (Kendall’s τ coefficient = 0.098, *p* = 0.003; Figure 5B) (James et al., 2019; Pottackal et al., 2020).

### Estimation of GABA_A_ Receptor Desensitization

A simulation was performed to estimate the potential impact of GABA_A_ receptor desensitization on the kinetics of IPSCs measured in DSGCs during WN stimulation of SACs. Each ON-OFF DSGC IPSC (response to full 100-s WN sequence) was first convolved with a peak-normalized exponential filter chosen to match the time course of fast GABA_A_ receptor desensitization (τ_decay_ = 35 ms) described by Jones and Westbrook (1995). Following convolution, the resulting trace was divided by the charge transfer of the mean emIPSC measured in DSGCs (1.4 pC per vesicle) and an estimate (500) of the average number of SAC synapses onto an ON-OFF DSGC (∼300–1000 synapses; Briggman et al., 2011; Sigal et al., 2015; Sethuramanujam et al., 2021). The resulting trace had units of vesicles per synapse, which were interpreted as the time-weighted mean number of vesicles interacting to drive fast desensitization at a single synapse at each point in time. A histogram was then computed from this time series. Finally, histograms were averaged across ON-OFF DSGCs (*n* = 10 cells; Figure 8).

### Statistics

Consistent with comparable studies, each experimental group comprised 4–10 cells from at least two mice of either sex. Unless otherwise stated, summary values are reported as mean ± SEM and statistical comparisons were performed using two-tailed Student’s *t*-tests. Exact *p*-values are reported up to *p* < 0.001. Statistical significance levels are indicated in figures as follows: ^∗^*p* < 0.05, ^∗∗^*p* < 0.01, and ^∗∗∗^*p* < 0.001.

## Results

### Computational Properties of GABAergic SAC Synapses Differ According to Postsynaptic Cell Type

To evaluate the possibility that GABAergic SAC synapses differ systematically according to postsynaptic cell type, we combined optogenetic stimulation of SACs with linear systems analysis of IPSCs evoked in SACs and DSGCs (Figure 1B). In each experiment, a local network of ChR2^+^ SACs was stimulated with a spot (400-μm diameter) of blue light (λ_peak_ = 470 nm) whose intensity was temporally modulated by a quasi-white-noise (WN) sequence (30-Hz cut-off; Pottackal et al., 2020). To block responses mediated by conventional photoreceptors (i.e., rods and cones), glutamate receptor drugs were applied throughout the recording period (see section “Materials and Methods”). Optogenetically evoked IPSCs recorded in either a SAC or a DSGC were quantified using LN cascade analysis, which generates a computational model consisting of two components: (1) a linear filter, which captures kinetic properties of the modeled synapse; and (2) a static nonlinearity, which captures time-independent properties of the synapse, including rectification and saturation (Figure 2A; Pottackal et al., 2020). Compared to a frequency-based analysis of a raw signal, an LN model-based analysis enables isolation of linear filtering from nonlinear properties. For example, a rectified signal features rapid changes above a threshold, which would increase the high-frequency content of the signal independently of any underlying linear filtering process. The time courses of IPSCs recorded in SACs and DSGCs were well-captured by LN models, which explained over 75% of the variance in an IPSC, on average (Figure 2B).

Using this paradigm, we compared GABAergic transmission at SAC synapses onto ON SACs and ON-OFF DSGCs. LN analysis revealed distinct computational properties in each postsynaptic cell type, such that low-pass filtering was significantly stronger in DSGCs than in SACs. Specifically, compared to linear filters obtained from ON SAC IPSCs, filters from ON-OFF DSGC IPSCs peaked later (25.2 ± 0.6 vs. 22.2 ± 0.5 ms; *p* = 0.003, *t* = 3.6) and were ∼70% wider (58.3 ± 2.2 vs. 33.5 ± 0.7 ms; *p* < 0.001, *t* = 10.9; Figures 2C,D,F). Additionally, comparison of static nonlinearities showed that IPSCs exhibited more rectification in ON-OFF DSGCs than in ON SACs (0.72 ± 0.05 vs. 0.51 ± 0.05; *p* = 0.012, *t* = 3.0; Figures 2C,D,F). We considered that the distinct temporal filters in the two cell types could reflect a difference in their synaptic input: ON-OFF DSGCs receive input from both ON and OFF SACs, whereas ON SACs receive input from ON, but not OFF, SACs (Briggman et al., 2011; Ding et al., 2016). We therefore studied ON DSGCs (Figures 2B,E), which receive input primarily from ON SACs (Yonehara et al., 2009; Dhande et al., 2013; Bae et al., 2018). Compared to IPSCs in ON-OFF DSGCs, those in ON DSGCs yielded similar filter peak times (26.6 ± 1.0 ms; *p* = 0.29, *t* = 1.1), filter widths (50.1 ± 3.0 ms; *p* = 0.052, *t* = −2.2), and rectification (0.62 ± 0.05; *p* = 0.25, *t* = −1.2; Figures 2C–E). Moreover, linear filters obtained from ON DSGC IPSCs were significantly delayed (*p* = 0.006, *t* = 3.8) and wider (*p* = 0.002, *t* = 5.3; Figures 2C,E,F) compared to those obtained from ON SAC IPSCs. These data suggest that computations performed by GABAergic SAC synapses are postsynaptic cell type-specific; i.e., they differ according to the postsynaptic cell type (SACs vs. DSGCs).

The above conclusions depend on the LN model to measure IPSC kinetics. To complement this approach, we compared the waveforms of IPSCs using two LN model-independent analyses. First, we computed normalized power spectra directly from each IPSC recording, which confirmed stronger low-pass filtering in IPSCs of ON-OFF DSGCs (5–35 Hz, *p* < 0.001) and ON DSGCs (10–35 Hz, *p* < 0.001) than in those of ON SACs (Supplementary Figure 1). Additionally, we computed the squared Pearson correlation coefficient *r*^2^) between each pair of IPSC recordings across cell types to test whether IPSCs were more similar within each group of postsynaptic cells (i.e., SACs or DSGCs) than between groups. For this analysis, *r*^2^ values were computed using the mean response to the repeated stimulus sequence in order to reduce noise in individual trials. The average *r*^2^ value within each group (SAC, ON-OFF DSGC, and ON DSGC) was >0.80, and the correlations within each DSGC group were similar to the correlations between DSGC groups (Supplementary Figure 1). By contrast, *r*^2^ values between SAC IPSCs and IPSCs of either DSGC type were significantly lower (SAC vs. ON-OFF DSGC: *r*^2^ = 0.58 ± 0.03, *p* < 0.001; SAC vs. ON DSGC: *r*^2^ = 0.61 ± 0.03, *p* = 0.003; Supplementary Figure 1). Thus, three distinct analytical approaches support the notion that computational properties of SAC synapses vary with postsynaptic cell type, with relatively strong low-pass filtering at synapses onto DSGCs.

### Fast Kinetics of IPSCs Measured in ChR2^+^ SACs Cannot Be Explained by Unclamped ChR2 Currents

We tested whether the relatively fast kinetics of IPSCs in a SAC could be explained by inadequate voltage-clamp, leading to distortive interactions between GABAergic and ChR2 currents in the recorded cell. Indeed, if the recorded SAC is not adequately voltage-clamped within compartments that contain both GABA_A_Rs and ChR2, co-activation of the associated conductances could systematically distort measurements of one or both conductances (Poleg-Polsky and Diamond, 2011). If this were the case, activation of one conductance could artificially accelerate the measured time course of the other conductance. Absent the ability to selectively block ChR2 in a recorded SAC while measuring IPSCs, we first evaluated this possibility by measuring ChR2 currents before and after blocking IPSCs (Figure 3A). WN-evoked ChR2 currents in SACs were equally reliable whether IPSCs were intact or blocked (*p* = 0.663, *t* = 0.47; Figure 3B). Critically, linear filters obtained from these currents were not prolonged under GABA_A_R blockade: filter peaks were not significantly delayed (*p* = 0.983, *t* = −3.1), and filter widths were not significantly increased (*p* = 0.896, *t* = −1.5, one-tailed Student’s *t*-tests; Figures 3C,D). The apparent capacity of voltage clamp to effectively null GABAergic currents in SACs is consistent with the particular spatial arrangement of inhibitory synapses onto SAC neurites in mice: a SAC receives a large majority of its inhibitory inputs on low-order neurites within 50 μm of its soma (Ding et al., 2016), relatively close to the recording site.

In contrast with inhibitory input synapses, ChR2 is distributed throughout the SAC arbor. In particular, because the distal tips of SAC neurites are likely under incomplete voltage clamp (Ding et al., 2016; Chen et al., 2020), the presence of ChR2 in these compartments could generate unclamped inward currents while recording IPSCs at the nominal E_cation_. To estimate whether contamination of GABAergic IPSCs by unclamped ChR2 currents could artificially accelerate IPSC kinetics, we performed a simulation (see section “Materials and Methods”). For this simulation, we considered the possibility that slow IPSCs recorded in ON-OFF DSGCs represent the “true” IPSC waveform which, in SACs, is distorted due to contamination by fast unclamped ChR2 currents (Figures 4A–C). However, when summed with (i.e., contaminated by) traces estimating unclamped ChR2 currents (see section “Materials and Methods”), IPSCs recorded in DSGCs did not increase in similarity to those recorded in SACs (Figures 4D,E). Although this analysis considers only linear interaction between IPSCs and unclamped ChR2 currents in SACs, several features of our experimental paradigm reduce the likelihood of nonlinear interactions mediated by voltage-gated ion channels (see section “Discussion”). Overall, the results in Figures 3, 4 suggest that imperfect voltage-clamp of SACs cannot explain the relatively fast kinetics of WN-evoked IPSCs recorded in SACs compared to DSGCs (Figure 2).

### Measurement and Comparison of Postsynaptic Filtering at GABAergic SAC Synapses

We next evaluated whether the specificity of temporal filtering at GABAergic SAC synapses could be explained by a postsynaptic mechanism, e.g., differential kinetics of GABA receptors on the postsynaptic cell types. To test this, we examined the unitary waveforms of IPSCs recorded from SACs and DSGCs to assess postsynaptic GABA receptor dynamics. We did not measure spontaneous miniature IPSCs (mIPSCs) for this purpose because both SACs and DSGCs receive additional inhibitory synapses from ACs other than SACs (Park et al., 2015; Pei et al., 2015; Ding et al., 2016; Bleckert et al., 2018; Huang et al., 2019). Instead, we briefly (<10 ms) stimulated ChR2^+^ SACs to evoke small, mIPSC-like events in ON SACs and DSGCs (Pottackal et al., 2020; Figure 5A), which we refer to as evoked monophasic IPSCs (emIPSCs). Following automated detection, sorting, and alignment of these emIPSCs, we measured individual and averaged emIPSCs recorded in both ON SACs and DSGCs.

Individual emIPSCs recorded from ON SACs (*n* = 401 events from 8 cells) and DSGCs (*n* = 212 events from 4 ON-OFF DSGCs and 1 ON DSGC; Pottackal et al., 2020) exhibited waveforms with similar decay kinetics (*p* = 0.075, *D* = 0.108, Kolmogorov–Smirnov test; Figure 5B). The emIPSC average in DSGCs, however, exhibited a prolonged “tail” during the decay phase, which was obscured by noise in individual emIPSCs but could be identified by a second, slow exponential decay term in the averaged waveform (τ_decay(fast)_ = 11.9 ms; τ_decay(slow)_ = 54.2 ms; Figure 5C; Pottackal et al., 2020). The ON SAC emIPSC average lacked this tail and therefore was well fit by a single exponential decay term (τ_decay_ = 18.1 ms; Figure 5C). These results suggest that postsynaptic mechanisms produce moderately stronger low-pass filtering for GABAergic transmission in DSGCs than in ON SACs.

### Pre- and Postsynaptic Contributions to Postsynaptic Cell Type-Specific Filtering at SAC Synapses

We next determined the extent to which differences in postsynaptic filtering (Figure 5) could explain overall differences in temporal filtering at SAC synapses (Figure 2). To this end, a Wiener deconvolution-based procedure was first used to estimate instantaneous presynaptic release rates from GABAergic IPSCs recorded in ON-OFF DSGCs during WN stimulation of presynaptic SACs (see section “Materials and Methods”; James et al., 2019; Pottackal et al., 2020). We then used the output of this procedure to generate “hybrid” IPSCs, which combined estimates of pre- and postsynaptic dynamics measured at the two types of GABAergic SAC synapse; i.e., the instantaneous presynaptic release rates at SAC→DSGC synapses were convolved with the postsynaptic filter (average emIPSC) at SAC→SAC synapses (Figure 6A). These hybrid IPSCs were then subjected to the LN analysis described above. If postsynaptic filtering (i.e., emIPSC waveform) suffices to explain postsynaptic cell type-specific filtering at SAC synapses, then the linear filters of hybrid IPSCs should match those of IPSCs with the same postsynaptic, rather than presynaptic, dynamics. Specifically, the wider (i.e., slower) linear filter for SAC→DSGC transmission should be “converted” to the narrower (i.e., faster) linear filter for SAC→SAC transmission.

We applied this analysis to test whether the moderate prolongation of the DSGC emIPSC compared to the SAC emIPSC (Figure 5C) could explain stronger low-pass filtering observed in SAC→DSGC IPSCs (Figure 2). This clearly was not the case, as linear filters extracted from SAC→SAC IPSC recordings were significantly narrower than those from hybrid IPSCs generated by combining SAC→DSGC presynaptic dynamics with SAC→SAC postsynaptic dynamics (*p* < 0.001, *t* = −8.8; Figures 6B–D). Filters from hybrid IPSCs were slightly (<5 ms) but significantly narrower than those from corresponding IPSCs recorded in ON-OFF DSGCs (*p* < 0.001, *t* = −15.3; Figures 6B–D); overall, however, differences in emIPSC waveform between GABAergic SAC→SAC and SAC→DSGC synapses play a relatively minor role in shaping the overall filtering properties revealed by LN analysis. Instead, the difference between filtering at these synapses appears to depend on distinct presynaptic properties.

To quantify postsynaptic cell type-specific temporal differences at SAC presynapses, we applied LN analysis directly to the estimates of instantaneous release rates extracted by Wiener deconvolution of IPSC recordings (Figures 7A–C). Strikingly, whereas presynaptic SAC→DSGC linear filters exhibited a monophasic waveform indicative of low-pass filtering (Figure 7B), presynaptic SAC→SAC linear filters exhibited a biphasic waveform indicative of band-pass filtering (Figure 7C). SAC→DSGC presynaptic filters were consistently wider (*p* < 0.001, *t* = 7.4; Figures 7B–D) and less biphasic (*p* < 0.001, *t* = −9.5; Figures 7B–D) than SAC→SAC presynaptic filters. Additionally, SAC→DSGC presynapses exhibited stronger rectification than SAC→SAC presynapses (*p* = 0.004, *t* = 3.7; Figures 7B–D). Independently of LN analysis, direct frequency analysis of the estimated instantaneous release rates confirmed the low- and band-pass characteristics of SAC presynapses onto DSGCs and SACs, respectively: whereas, on average, normalized power spectra of SAC→DSGC presynapses decreased monotonically with frequency, those of SAC→SAC presynapses peaked near 20 Hz and, overall, more effectively passed frequencies between 10 and 35 Hz (*p* < 0.001; Figure 7E). Collectively, these analyses suggest that SAC presynapses exhibit distinct computational properties that co-vary with the identity of the postsynaptic cell type.

Because fast desensitization of GABA_A_ receptors can prolong receptor deactivation and the decay of macroscopic IPSCs (Jones and Westbrook, 1995; Haas and Macdonald, 1999), an alternative explanation for postsynaptic cell type-specific GABAergic transmission is that vesicle release at individual SAC→DSGC synapses strongly drives fast GABA_A_ receptor desensitization, which in turn prolongs IPSC decay. Specifically, fast desensitization (τ∼ 10’s of ms) drives IPSC prolongation by increasing the relative contribution of the slower component of biexponential IPSC decay (Jones and Westbrook, 1995; Haas and Macdonald, 1999; Bianchi et al., 2007). To gain insight into whether postsynaptic receptor desensitization at SAC→DSGC synapses could explain the relatively strong filtering at these synapses, we analyzed IPSCs recorded in ON-OFF DSGCs during WN stimulation of SACs. By combining these IPSCs with measurements of single-vesicle charge transfer and estimated counts of SAC input synapses to a DSGC (see section “Materials and Methods”), we estimated the average number of vesicles per synapse interacting over a time scale typical of fast GABA_A_ receptor desensitization (τ = 35 ms; Jones and Westbrook, 1995). The analysis yielded an estimated median of 0.005 (interquartile range: 0.002–0.013) vesicles per synapse interacting *via* fast desensitization at any given time (Figure 8). This result suggests that vesicle release at an individual SAC synapse occurs at a low rate in our WN paradigm, likely rendering fast GABA_A_ receptor desensitization negligible. Thus, we conclude that postsynaptic GABA_A_ receptor desensitization is highly unlikely to account for postsynaptic cell type-specific transmission at SAC synapses.

### Distinct VGCC Populations Mediate Release From SAC→DSGC and SAC→SAC Presynapses

**FIGURE 9:**
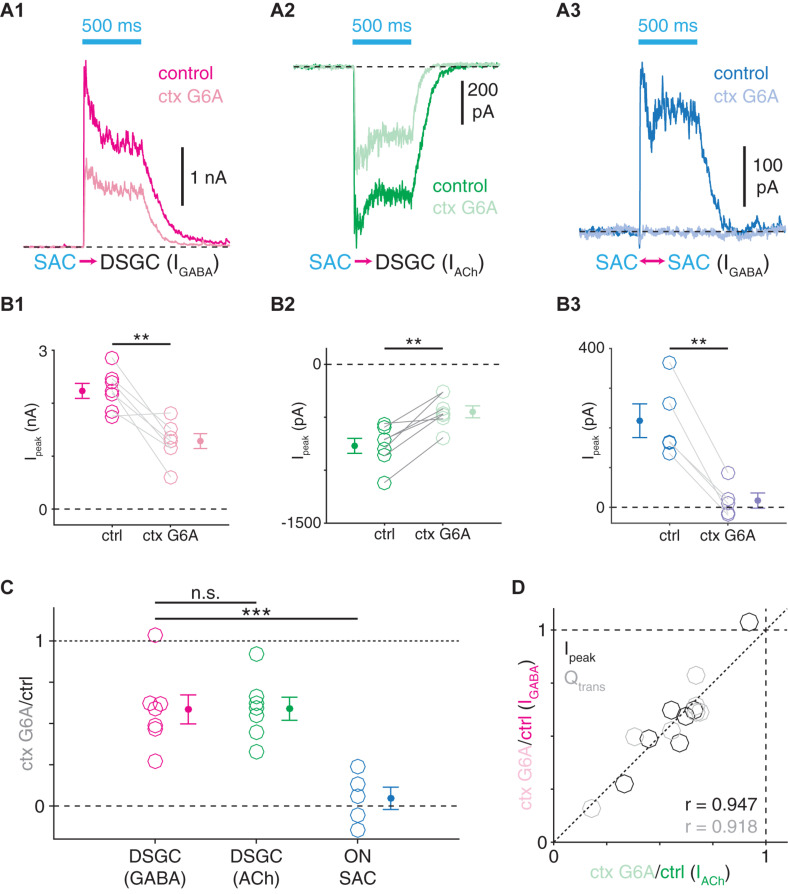
Distinct VGCC populations mediate transmitter release from SAC synapses onto DSGCs and SACs. **(A)** ChR2-evoked IPSCs **(A1)** and EPSCs **(A2)** in ON-OFF DSGCs and IPSCs in ON SACs **(A3)** before and during bath application of ω-conotoxin G6A (ctx G6A, 300 nM), an N-type VGCC blocker. **(B)** Peak amplitudes (I_peak_) of ChR2-evoked IPSCs (**B1**, *n* = 7) and EPSCs (**B2**, *n* = 7) in ON-OFF DSGCs and IPSCs in ON SACs (**B3**, *n* = 5) before and during ω-conotoxin G6A application. **(C)** Residual fractions of peak amplitude following ω-conotoxin G6A application. **(D)** ω-conotoxin G6A sensitivities of ChR2-evoked GABAergic IPSCs (I_GABA_) and cholinergic EPSCs (I_ACh_) measured in the same ON-OFF DSGCs (*n* = 7). Residual fractions of peak amplitude (I_peak_) or charge transfer (Q_trans_) during ctx G6A application. n.s., not significant; **p < 0.01; ***p < 0.001.

**FIGURE 10:**
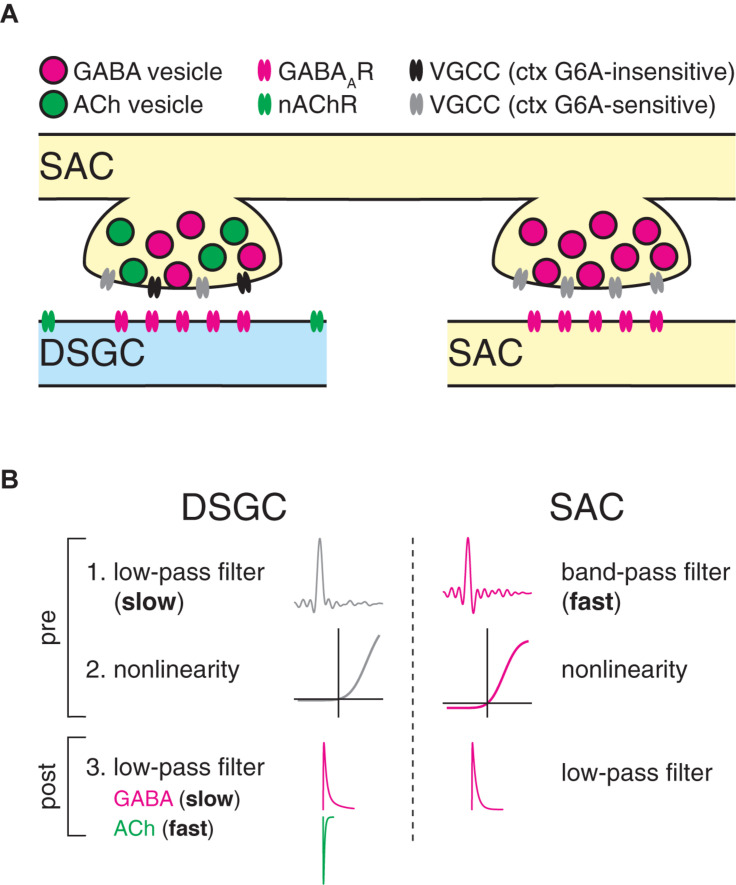
Proposed model for postsynaptic cell type-specific computation at GABAergic SAC synapses. **(A)** Summary diagram illustrating distinct molecular composition of SAC output synapses. Two neighboring output varicosities on a presynaptic SAC neurite participate in separate synapses with a direction-selective ganglion cell dendrite (DSGC, blue) and a SAC neurite (SAC, yellow). Synaptic transmission onto DSGCs is mediated by a heterogeneous population of VGCCs. Extrasynaptic localization of nicotinic ACh receptors reflects a presumed paracrine mode of ACh transmission (Pottackal et al., 2020; Sethuramanujam et al., 2021). By contrast, synaptic transmission onto SACs is mediated by a homogenous population of VGCCs. **(B)** Computational description of SAC synapses. At DSGC synapses, SAC presynapses perform low-pass filtering with relatively strong rectification, followed by additional low-pass filtering by postsynaptic mechanisms. Postsynaptic low-pass filtering is stronger for GABAergic transmission than for cholinergic transmission. By contrast, at synapses onto SACs, SAC presynapses perform fast band-pass filtering with milder rectification, followed by additional postsynaptic low-pass filtering.

The analyses above support a model wherein the transmission dynamics of GABAergic SAC presynapses correspond with postsynaptic cell type, suggesting that synaptic protein expression could likewise vary between SAC presynapses (Cohen, 2001; Lee et al., 2010). We tested this possibility by determining whether the same VGCCs mediate neurotransmitter release from SAC presynapses onto SACs and DSGCs. The N-type VGCC antagonist ω-conotoxin G6A (ctx G6A) reduced peak amplitudes of ChR2-evoked PSCs for all of three cases: IPSCs in ON-OFF DSGCs (*p* = 0.004, *t* = −4.6), EPSCs in ON-OFF DSGCs (*p* = 0.002, *t* = 5.5), and IPSCs in ON SACs (*p* = 0.003, *t* = −6.5; Figures 9A,B). However, whereas ChR2-evoked IPSCs (41.5 ± 8.8%) and EPSCs (41.1 ± 7.0%) in ON-OFF DSGCs were similarly and only partially reduced (*p* = 0.89, *t* = −0.15; Figure 9C), IPSCs were far more severely reduced in ON SACs (95.3 ± 6.8%) than in ON-OFF DSGCs (*p* < 0.001, *t* = 4.9; Figure 9C). Ctx G6A sensitivities of cholinergic and GABAergic transmission at SAC→DSGC synapses were highly correlated in individual cells (PSC peak amplitude: *r* = 0.95, *p* = 0.001; PSC charge transfer: *r* = 0.92, *p* = 0.004; Figure 9D), supporting a model in which ACh and GABA are released at the same presynapses onto DSGCs (Figure 10A; Pottackal et al., 2020). Thus, transmission at SAC→DSGC and at SAC→SAC synapses rely on distinct VGCC populations, with either partial (DSGC) or near-complete (SAC) dependence on N-type VGCCs. Interestingly, fluorescence imaging of ACh at varicosities of individual SACs suggests that only a subset of varicosities detectably release ACh (Sethuramanujam et al., 2021), which could in principle reflect a subpopulation of SAC output synapses that release only GABA onto SACs (Figure 10A).

Differential VGCC expression, however, does not appear to explain postsynaptic cell type-specific filtering at GABAergic SAC presynapses, as LN analysis of IPSCs recorded in ON-OFF DSGCs during N-type VGCC blockade yielded linear filters with widths similar to controls (*p* = 0.45, *t* = −0.80; Supplementary Figure 2). Likewise, the sensitivity of ChR2-evoked IPSCs to varying extracellular (Ca^2+^) did not differ in ON SACs and ON-OFF DSGCs (Supplementary Figure 3), suggesting that functionally similar calcium-sensing proteins mediate release at SAC presynapses onto each cell type. Thus, it is likely that additional, unidentified molecules are differentially expressed within SAC presynapses to enable postsynaptic cell type-specific computations. Though the precise molecular correlates of postsynaptic cell type-specific filtering remain to be elucidated, overall, the apparently heterogeneous profiles of VGCC expression in SAC synaptic terminals support the more general notion that properties of SAC presynapses vary in a systematic (i.e., postsynaptic cell type-specific) manner.

## Discussion

We investigated parallel computation at outputs of the SAC, a retinal interneuron that makes closely spaced synapses—from the same neurite and separated by only a few micrometers—onto neighboring SACs and DSGCs (Ding et al., 2016). Within small regions of SAC neurite, [Ca^2+^] changes uniformly in response to visual stimulation (Poleg-Polsky et al., 2018). At a finer scale, however, the dynamics of GABAergic transmission differ according to the postsynaptic cell type: specifically, dynamics at SAC→SAC synapses are accelerated compared to those at SAC→DSGC synapses (Figure 2). Our experimental and computational analyses suggest that this difference arises primarily at a pre- rather than postsynaptic locus (Figures 5–8). Thus, adjacent SAC presynapses likely act as distinct temporal filters that differ with the identities of their postsynaptic cell types (Figure 10B).

### Candidate Mechanisms for Postsynaptic Cell Type-Specific Filtering at GABAergic SAC Synapses

Temporal filtering at a synapse depends, in part, on the initial release probability (P_*r*_) of vesicles docked at the release site: high P_*r*_ generally promotes low-pass filtering, arising from short-term depression; whereas low P_*r*_ generally promotes high- and band-pass filtering, arising from a combination of short-term facilitation and depression (Zucker and Regehr, 2002; Abbott and Regehr, 2004; Körber and Kuner, 2016). Initial P_*r*_ depends on mechanisms including the presynaptic Ca^2+^ sensor and associated proteins, biophysical properties of presynaptic VGCCs, and VGCC-release site coupling (Stanley, 2016; Kaeser and Regehr, 2017; Jackman and Regehr, 2017; Dittman and Ryan, 2019). Differences in P_*r*_ could distinguish SAC→DSGC and SAC→SAC presynapses: for example, our results predict that initial P_*r*_ would be higher at SAC→DSGC presynapses (low-pass filtering) than at SAC→SAC presynapses (band-pass filtering) (Figure 7). The differences in temporal filtering we observed at the two types of SAC presynapse, however, do not appear to depend on differential properties of either VGCCs (Supplementary Figure 2) or calcium-sensing proteins (Supplementary Figure 3).

Additionally, postsynaptic cell type-specific differences in the geometric volume of individual SAC presynapses could contribute to distinct temporal filtering profiles, as has been observed at axon terminals of single bipolar cells (BCs) in zebrafish retina (Baden et al., 2014). In these BCs, large terminals exhibit low-pass filtering, whereas smaller terminals tend toward band-pass filtering. If presynaptic geometry similarly tunes temporal filtering at SAC synapses, we would predict that stronger low-pass filtering at SAC→DSGC presynapses associates with a larger geometric volume. Interestingly, SAC→DSGC presynaptic varicosities characteristically wrap around postsynaptic DSGC dendrites (Yamada et al., 2003; Briggman et al., 2011), which could in turn increase terminal volume. The geometry of SAC→SAC synapses has not been described as extensively; consequently, additional ultrastructural analysis will be required to evaluate whether postsynaptic cell type-specific differences in terminal volume could reliably shape temporal filtering at SAC synapses.

In theory, postsynaptic cell type-specific synaptic filtering could arise from a circuit-level mechanism, i.e., distinct presynaptic inhibitory input to the different types of SAC presynapse. This seems unlikely, however, because inhibitory inputs are localized almost exclusively to the proximal third of SAC neurites, near the soma; whereas SAC output synapses are localized to the distal third of neurites, near their tips (Ding et al., 2016). This stands in contrast to axon terminals of single BCs in salamander retina, where inhibitory inputs target subsets of presynaptic release sites and apparently can differentiate release at individual synapses (Asari and Meister, 2012).

We also identified a modest prolongation of postsynaptic GABA_A_R-mediated current decay kinetics at SAC→DSGC synapses compared to those at SAC→SAC synapses (Figure 5); this difference in kinetics could reflect differential expression and/or localization of GABA_A_R subunits in DSGCs and SACs (Brandstätter et al., 1995; Auferkorte et al., 2012). Analogously, at glutamatergic synapses from cone photoreceptors to distinct OFF BC types, postsynaptic cell type-specific expression of AMPA and kainate receptors enables differential filtering of cone input (DeVries, 2000; Puller et al., 2013; Lindstrom et al., 2014; Ichinose and Hellmer, 2016; but see Borghuis et al., 2014; Puthussery et al., 2014).

### Potential Consequences for Retinal Direction-Selective Circuit Function

Direction selectivity first emerges in the DS circuit at the level of GABA release from SACs. The DS tuning of GABA release depends on several mechanisms, including the spatiotemporal integration of excitatory input from presynaptic BCs, intrinsic properties of SAC neurites, and inhibitory synapses onto SACs (Euler et al., 2002; Lee and Zhou, 2006; Hausselt et al., 2007; Kim et al., 2014; Vlasits et al., 2016; Ding et al., 2016; Chen et al., 2016; Fransen and Borghuis, 2017). DS tuning is subsequently transferred to the DSGC by asymmetric wiring at GABAergic SAC→DSGC synapses: a DSGC that prefers motion in one direction (e.g., leftward) receives GABAergic synapses selectively from SAC neurites that prefer motion in the opposite direction (e.g., rightward) (Fried et al., 2002; Lee et al., 2010; Wei et al., 2011; Briggman et al., 2011; Morrie and Feller, 2015).

We observed that GABAergic SAC→DSGC synapses exhibit stronger low-pass filtering than GABAergic SAC→SAC synapses (Figure 2), primarily due to band-pass filtering at SAC→SAC presynapses (Figure 7). Functionally, SAC→DSGC inhibition serves to “veto” excitation during null-direction motion and, thus, acts critically to shape the DS spike output of DSGCs. In this context, relatively prolonged kinetics would enable inhibition to fully outlast coincident excitatory input to DSGCs generated by glutamate release from BCs and ACh release from SACs. By contrast, SAC→SAC inhibition appears to be dispensable for generating direction selectivity in SAC neurites (Chen et al., 2016). Instead, SAC→SAC inhibition appears to promote direction selectivity in DSGCs by transiently hyperpolarizing SACs, thereby relieving synaptic depression at GABAergic SAC→DSGC synapses in response to motion on a noisy background (Chen et al., 2020). Milder low-pass filtering at SAC→SAC synapses, then, may enable the relief of synaptic depression at SAC→DSGC synapses over a relatively wide range of stimulus velocities.

### Optogenetically Evoked IPSC Recordings in SACs Are Minimally Impacted by ChR2 Expression

To study inhibitory synaptic input to SACs, we recorded optogenetically evoked IPSCs in cells that expressed ChR2. In theory, the ChR2 current should have been neutralized by clamping the membrane potential at E_cation_. Given the complex geometry of the SAC dendritic tree, however, we considered the presence of an unclamped ChR2 current and its possible impact on the measurement of IPSC kinetics. In our analysis (Figure 4), we assume that the interaction between unclamped ChR2 currents and IPSCs in SACs is linear, despite the presence of both voltage-gated sodium channels (VGSCs) and VGCCs in these cells (Cohen, 2001; Kaneda et al., 2007; O’Brien et al., 2008; Lee et al., 2010; Oesch and Taylor, 2010; Koren et al., 2017; Pottackal et al., 2020). In principle, activation of ChR2 in poorly clamped distal neurites could generate non-linear interactions with VGSCs and/or VGCCs. However, for multiple reasons, it seems likely that both VGSCs and VGCCs are effectively neutralized under our recording conditions. First, we included in the recording pipette the VGSC blocker QX-314, which effectively blocks the tetrodotoxin-resistant Na_v_ 1.8 type of VGSC (Leffler et al., 2005; Johansson et al., 2019) that is expressed by SACs (O’Brien et al., 2008; Oesch and Taylor, 2010). In support of this point, current-voltage relationships measured in mouse SACs loaded with 2–3 mM QX-314 are highly linear (Stincic et al., 2016; Fransen and Borghuis, 2017). Second, voltage-clamp of SACs at depolarized potentials (>−30 mV) for 1–10 s is sufficient to abolish regenerative currents generated by VGCCs (Fransen and Borghuis, 2017; Koren et al., 2017), presumably *via* voltage-dependent inactivation. By comparison, in our paradigm, SACs were clamped near E_cation_ for >120 s. Although GABA_A_ receptors in distal neurites could provide sufficient hyperpolarization to deinactivate local VGCCs, there is only sparse spatial overlap between synaptic outputs and inhibitory synaptic inputs in SAC neurites (Ding et al., 2016), decreasing the likelihood that such VGCC deinactivation is prevalent. Moreover, because ChR2 stimulation alone is sufficient to drive VGCC-dependent release from SACs (Sethuramanujam et al., 2016; Hanson et al., 2019; Pottackal et al., 2020), it seems plausible that the concerted action of local ChR2 activation and somatic current injection would sufficiently depolarize distal SAC neurites to prevent VGCCs from deinactivating appreciably. Overall, our analysis shows that any impact of unclamped ChR2 current in SACs cannot explain the relatively fast kinetics of IPSCs in response to WN stimulation (Figures 3, 4).

## Conclusion

Retinal interneurons feature several mechanisms for parallel processing at unusually fine spatial scales. For example, output synapses from single, narrow-field retinal interneurons can exhibit functional diversity (Asari and Meister, 2012; Baden et al., 2014; Lee et al., 2016; Tien et al., 2016). In these cases, a key question is whether diverse synaptic outputs map systematically onto diverse postsynaptic cell types. Indeed, at least one retinal interneuron uses either glycine or glutamate at its output synapses in a postsynaptic cell type-specific manner (Lee et al., 2016; Tien et al., 2016). The present study builds conceptually upon this work by demonstrating that postsynaptic cell type-specific differences in synaptic computation can be implemented presynaptically, likely by intrinsic mechanisms. Finally, our work also suggests that the SAC combines at least two distinct parallel processing strategies, operating over different spatial scales, to diversify its output: (1) functional compartmentalization of its neurites (Euler et al., 2002; Koren et al., 2017; Poleg-Polsky et al., 2018; Morrie and Feller, 2018) and (2) postsynaptic cell type-specific filtering at synaptic terminals within each compartment (Figure 10).

## Data Availability Statement

The raw data supporting the conclusions of this article will be made available by the authors, without undue reservation.

## Ethics Statement

The animal study was reviewed and approved by the Institutional Animal Care and Use Committee at Yale University.

## Author Contributions

JP, JS, and JD: conceptualization, methodology, and writing – review and editing. JP and JD: software. JP: formal analysis, investigation, writing – original draft, and visualization. JD: supervision. JS and JD: funding acquisition. All authors contributed to the article and approved the submitted version.

## Conflict of Interest

The authors declare that the research was conducted in the absence of any commercial or financial relationships that could be construed as a potential conflict of interest.

## Publisher’s Note

All claims expressed in this article are solely those of the authors and do not necessarily represent those of their affiliated organizations, or those of the publisher, the editors and the reviewers. Any product that may be evaluated in this article, or claim that may be made by its manufacturer, is not guaranteed or endorsed by the publisher.
