# Supplementary material for: Computational and Molecular Properties of Starburst Amacrine Cell Synapses Differ With Postsynaptic Cell Type
Source: Front Cell Neurosci. 2021 Jul 26;15:660773. doi: 10.3389/fncel.2021.660773 (PMC8351878; doi:10.3389/fncel.2021.660773)
Supplement: Supplementary file 1 [file Data_Sheet_1.pdf]

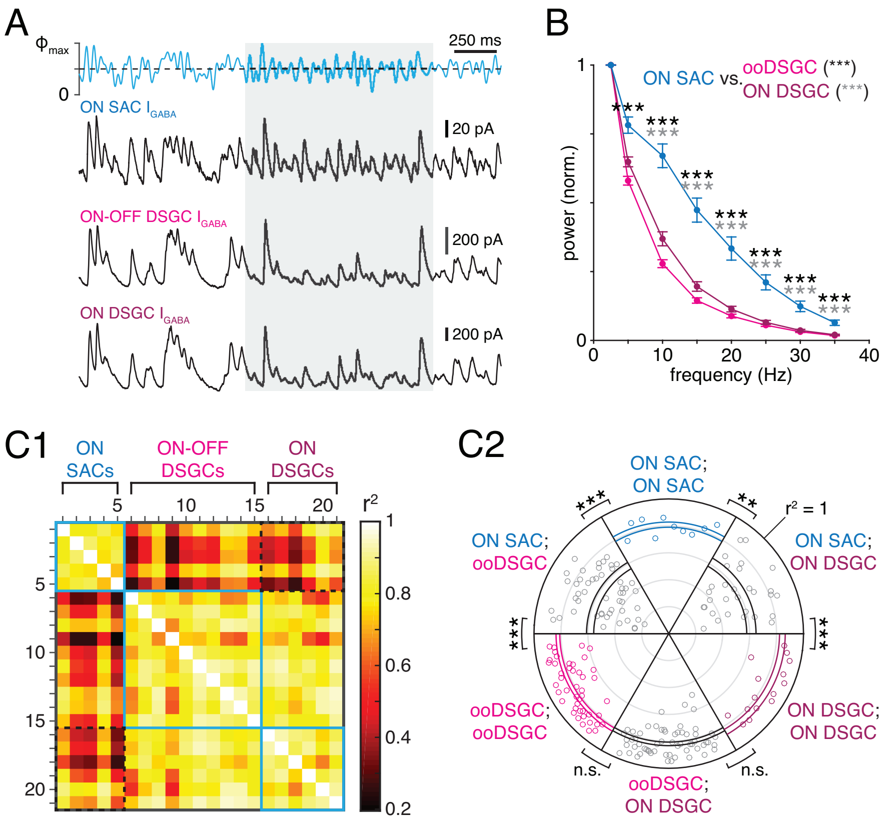

FIGURE S1 Model-independent evidence for postsynaptic cell type-specific computation at GABAergic SAC synapses. **(A)** IPSCs recorded and averaged across 10 trials in an ON SAC, an ON-OFF DSGC, and an ON DSGC during optogenetic WN stimulation of SACs. Gray box highlights stimulus period that more robustly evokes IPSCs in ON SACs than in DSGCs. **(B)** Frequency analysis of WN IPSCs recorded in ON SACs and DSGCs. Prior to averaging across cells, each power spectrum was normalized to its power at 2.5 Hz. Power spectra reflect the entire 100-s duration of each WN IPSC recording. Data from ON-OFF DSGCs are re-plotted from Pottackal et al. (2020). **(C)** Cell type-dependence of correlations between individual IPSC recordings. **C1**, Symmetric matrix of squared Pearson correlation coefficients (r^2) between all pairs of WN IPSC recordings. Groups of within-cell-type correlations are indicated by cyan boxes along the diagonal, while groups of between-cell-type correlations are separated by dashed black lines and lie off the diagonal. r^2 values are computed from averaged responses to the repeated stimulus only. **C2**, Summary of r^2 values used to construct matrix shown in **C1**. Within each group/sector, pairs of colored (within-type) or black (between-type) arcs indicate ± SEM across cells. The angle of each point within a group/sector is arbitrary.

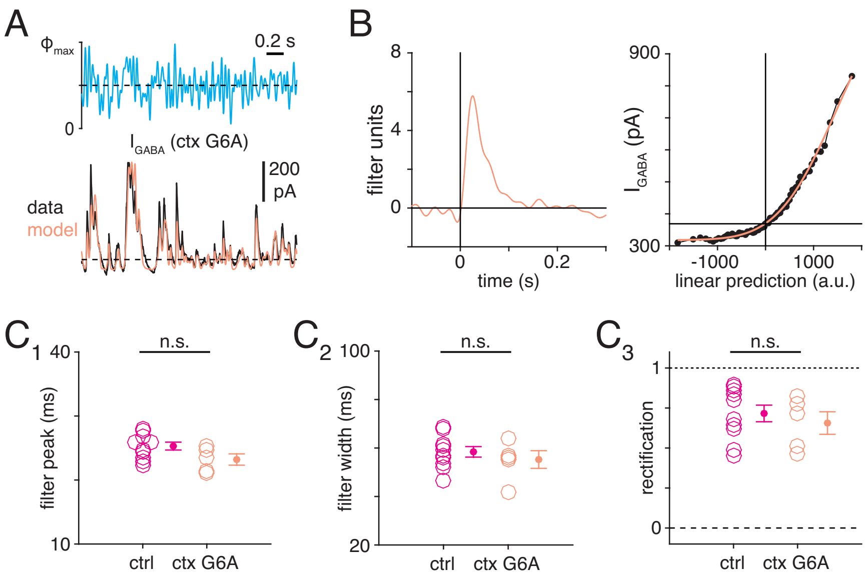

FIGURE S2 IPSCs mediated exclusively by non-N-type VGCCs are similar to control IPSCs in DSGCs. **(A)** Linear-nonlinear model of residual ChR2-evoked IPSCs in an ON-OFF DSGC during blockade of N-type VGCCs by ω-conotoxin G6A (ctx G6A, 300 nM). Model fit: r^2 = 0.858 ± 0.008 (n = 5). **(B)** Linear filter (*left*) and static nonlinearity (*right*) obtained from recording shown in **A**. **(C)** Measurements of LN model components obtained from IPSCs recorded in the absence (ctrl, n = 10) or presence (ctx G6A, n = 5) of ω-conotoxin G6A.

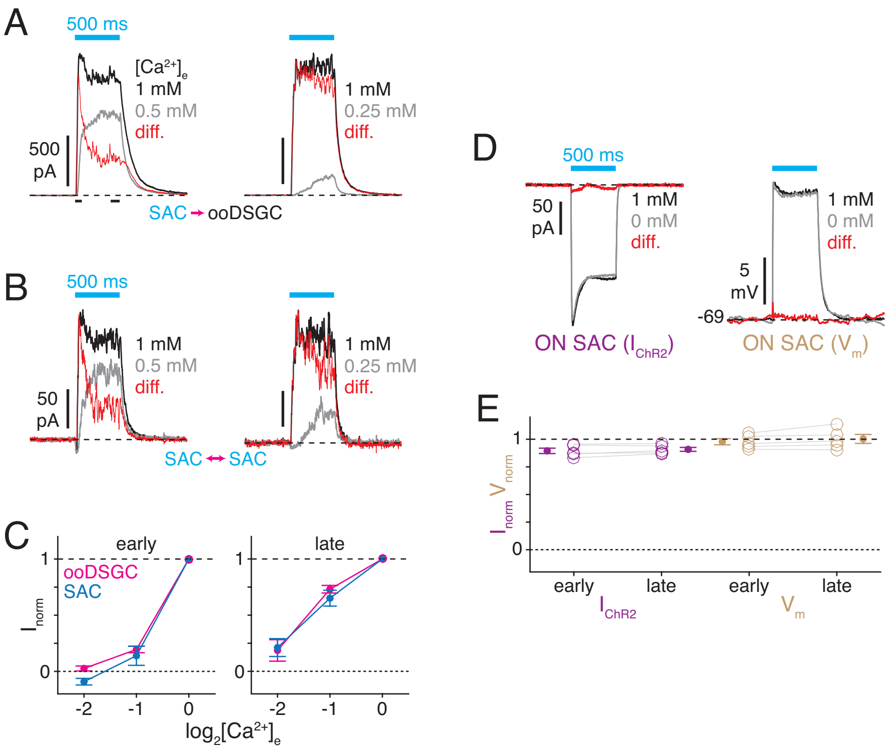

FIGURE S3 The calcium dependence of GABAergic transmission from SACs does not differ with postsynaptic cell type. **(A)** Sensitivity of ChR2-evoked IPSCs recorded in separate ON-OFF DSGCs to two- (*left*) and four-fold (*right*) reductions in extracellular Ca^{2+} concentration ($[Ca^{2+}]_e$). *Left*, Black bars indicate early and late response periods quantified in **C**. For each cell, red trace indicates difference between 1 mM and reduced $[Ca^{2+}]_e$ conditions. Stimulus intensity, 4.8×10^{17} Q cm^{-2} s^{-1}. **(B)** Same as **A** for ON SACs. **(C)** Normalized IPSC amplitudes during early (*left*) and late (*right*) response periods following two- and four-fold reductions in $[Ca^{2+}]_e$ for both ON-OFF DSGCs (magenta, n = 4, 4) and ON SACs (blue, n = 5, 5). **(D)** Sensitivity of ChR2-mediated photocurrent (*left*) and depolarization (*right*) in separate ON SACs to removal of extracellular Ca^{2+} (i.e., $[Ca^{2+}]_e$ = 0 mM). **(E)** Normalized ChR2-mediated photocurrent (*left*, n = 5) and depolarization (*right*, n = 5) in ON SACs following removal of extracellular Ca^{2+}.
